# Supplementary material for: Causal Relationship between Aging and Anorexia Nervosa: A White-Matter-Microstructure-Mediated Mendelian Randomization Analysis
Source: Biomedicines. 2024 Aug 16;12(8):1874. doi: 10.3390/biomedicines12081874 (PMC11351342; doi:10.3390/biomedicines12081874)
Supplement: Supplementary file 1 [file biomedicines-12-01874-s001.zip › Supplementary Figure/Figure S8/Leaveoneout plot of MR analysis for TL to ubm-b-1905.pdf]

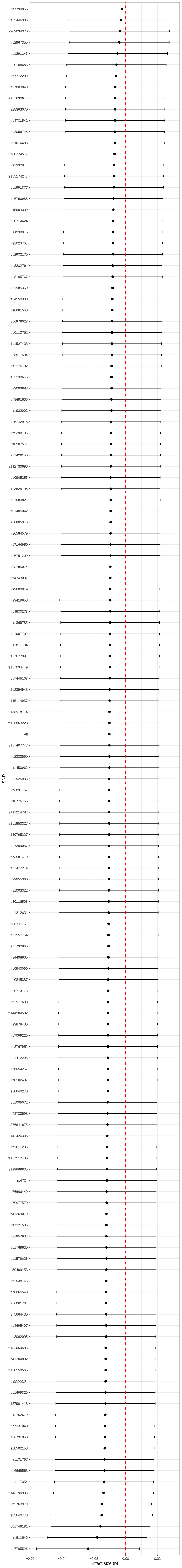
rs7790856
rs35446936
rs202034370
rs2967355
rs1291143
rs10768683
rs7772289
rs17803849
rs117630647
rs28363070
rs4731541
rs2056726
rs4616688
rs80324517
rs1332941
rs185174247
rs11991877
rs6790988
rs45604339
rs10774624
rs939916
rs1023767
rs12932179
rs2282764
rs8105767
rs1985369
rs34550383
rs6881568
rs16978028
rs10112752
rs111527438
rs28577594
rs2276182
rs13230646
rs3093888
rs78491606
rs932002
rs5742915
rs9398196
rs6587577
rs11426156
rs142730696
rs10905255
rs113525195
rs11584821
rs61405042
rs10805346
rs6584579
rs7164950
rs6751209
rs3785074
rs4743037
rs9600019
rs9419958
rs4530278
rs869785
rs1907702
rs871134
rs17677991
rs117034449
rs17445108
rs112394943
rs145114957
rs188918174
rs116863223
All
rs117407747
rs2230590
rs450962
rs12925933
rs3891167
rs6776756
rs141214782
rs111950327
rs139795227
rs7209057
rs73581419
rs12412214
rs9955360
rs1003322
rs80116508
rs11212631
rs55747751
rs11557154
rs77732866
rs4498805
rs9940099
rs10845387
rs10773176
rs2977608
rs144204502
rs9878436
rs7099229
rs3767952
rs11412296
rs6054257
rs8102497
rs10840270
rs11085072
rs73730598
rs376641875
rs142426306
rs1611236
rs117512405
rs139669835
rs4724
rs76666449
rs79977579
rs41269079
rs7221585
rs1957937
rs11769630
rs11579626
rs59409453
rs2538745
rs76065543
rs56061761
rs75664430
rs4695407
rs13062095
rs182059586
rs41304832
rs150150565
rs2555104
rs11699829
rs137901416
rs762679
rs77231040
rs66731853
rs28502153
rs131797
rs6669563
rs11117354
rs143190905
rs2763979
rs35640778
rs61748181
rs611646
rs7705526
SNP
-0.06
-0.04
-0.02
0.00
0.02
Effect size (b)
